# Female nurses have a higher prevalence of urinary tract symptoms and infection than other occupations in dialysis units

**DOI:** 10.1590/2175-8239-JBN-2020-0248

**Published:** 2021-05-21

**Authors:** Fabiana B Nerbass, Cintia E Santo, Edilaine V Fialek, Viviane Calice-Silva, Marcos A Vieira

**Affiliations:** 1 Fundação Pró-Rim, Joinville, SC, Brasil.

**Keywords:** Nurses, Hydration, Urinary Tract Infection, Urinary Tract Symptom, Toilet Behavior, Occupational Health, Técnicas de Enfermagem, Hidratação, Infecções Urinárias, Sintoma do Trato Urinário, Comportamentos Relacionados com a Saúde, Saúde do Trabalhador

## Abstract

**Introduction:**

Urinary tract symptoms and infection have been associated with occupational factors that impact hydration habits particularly in women. We compared self-reported urinary symptoms and infection and hydration habits between nurses and other occupations in dialysis units.

**Methods:**

Cross-sectional study. Participants worked in five nephrology centers in Brazil and answered an online questionnaire comprising questions regarding urinary tract symptoms and infection episodes in the preceding year; data on usual daily beverage intake, urine frequency, and urine color according to a urine color chart were also collected, as well as perceptions of water access and toilet adequacy at work.

**Results:**

We included 133 women (age=36.9±9.5 years). The self-reported usual daily beverage intake was 6.6±2.9 cups/day (~1320 mL), daily urine frequency was 5.4±2.1, and urine color chart score: 3.0±1.2. Nurses (N=66/49.6%) reported higher prevalence of burning sensation (50 versus 27%; P<0.001), urinary urgency (42 versus 21%; P<0.001), and infection (42% versus 25%; P=0.04) as well as lower liquid intake (6.0±2.6 versus 7.3±3.0 cups/day; P=0.01) than controls. Forty four percent of nurses reported being able to drink when thirsty "always" and "most of the time" versus 93% of the control group.

**Conclusion:**

Dialysis female nurses reported lower beverage intake and higher prevalence of symptoms and infection than other occupations in the same environment. Interventions to improve hydration can potentially decrease urinary problems in this population.

## Resumo

**Introdução:**

Sintomas e infecções do trato urinário têm sido associados a fatores ocupacionais que afetam hábitos de hidratação, particularmente em mulheres. Comparamos sintomas e infecções urinárias autorrelatados e hábitos de hidratação entre técnicas de enfermagem e outras ocupações em unidades de diálise.

**Métodos:**

Estudo transversal. As participantes trabalhavam em cinco centros de nefrologia no Brasil e responderam um questionário online contendo perguntas sobre sintomas do trato urinário e episódios de infecção no ano anterior; também foram coletados dados sobre a ingestão diária habitual de bebidas, frequência e cor da urina de acordo com uma tabela de cores da urina, assim como percepções sobre o acesso à água e adequação das instalações sanitárias no trabalho.

**Resultados:**

Incluímos 133 mulheres (idade=36,9±9,5 anos). Em média, as participantes relataram ingestão de bebidas de 6,6±2,9 copos/dia (~1320 mL), frequência urinária de 5,4±2,1 vezes/dia e escore da tabela de cores da urina de 3,0±1,2. Técnicas de enfremagem (N=66/49,6%) relataram maior prevalência de sensação de ardor (50 versus 27%; P<0,001), urgência urinária (42 versus 21%; P<0,001), e infecção (42% versus 25%; P=0,04), bem como menor ingestão de líquidos (6,0±2,6 versus 7,3±3,0 copos/dia; P=0,01) do que controles. Quarenta e quatro por cento das técnicas de enfermagem relataram poder beber quando têm sede "sempre" e "na maioria das vezes" contra 93% do grupo controle.

**Conclusão:**

Técnicas de enfermagem de unidades de diálise relataram menor ingestão de bebidas e maior prevalência de sintomas e infecção do que outras ocupações no mesmo ambiente de trabalho. Intervenções para melhorar a hidratação podem diminuir problemas urinários nesta população.

## Introduction

In practice, staying well hydrated means drinking sufficient water during the day and emptying the bladder whenever required^1^. Although seemingly simple, human hydration is complex and is influenced by physiological, dietary, metabolic, environmental, and behavioral factors^2^.

In recent years, the relationship between hydration and several health problems have gained increasing attention. Urinary tract symptoms and infection have been associated with decreased water intake and unhealthy toilet behaviors, particularly in women^3^^,^ 4.

While urinary symptoms can compromise the quality of life and productivity^5^, urinary tract infections are associated with high antibiotic prescriptions^6^ and an increased risk of antibiotic-resistant urinary pathogens^7^.

Although still scarcely explored among women who work, reports showed a higher prevalence of urinary issues in teachers^8^, brick workers^9^, field workers^9^, clean-room workers^3^, and, especially, nurses^4^^,^ 10 ^,^ 11. Occupational factors linked to urinary disorders include highly demanding jobs, lack of breaks, poor or lack of toilet facilities, use of special clothes, warm environments, and employment restrictions^3^^,^ 8 ^-^ 13. Among restrictions, the prohibition of having water bottles in hemodialysis rooms due to infection control regulations and the impossibility of leaving patients unattended are common rules in dialysis units. Although this topic has not been explored in these settings, we hypothesize that these regulations can impact hydration habits and urinary issues of female dialysis nurses.

In this cross-sectional multicenter study, we compared the prevalence of self-reported urinary symptoms and infections and hydration markers between dialysis nurses and other occupations sharing the same work environment.

## Methods

### Participants and setting

All female workers employed in five nephrology centers located in four cities in Santa Catarina State (Southern Brazil) were invited to participate (n=258). This analysis included workers employed for at least 12 months who were not pregnant or breastfeeding in the last year and with weekly work shifts varying between 30 to 42 hours over five or six days per week. We divided our population into two groups: the nurse and control groups. The nursing staff comprised workers who performed their activities in dialysis rooms. Their 7.5-hour workday included two breaks for meals (lasting 15 and 30 min). Toilets and water supplies were available outside of the dialysis rooms (due to infection control regulations, water bottles are not permitted in the nurse workstations). Because of patient care assistance, nurses cannot leave the dialysis rooms for extra breaks to drink water or use the toilet without assigning a substitute.

The control group comprised administrative and multidisciplinary staff who are permitted to have water bottles in their workstations. Although some workers from the multidisciplinary team spend part of the day in the dialysis rooms, they have a workstation outside of these rooms in which water bottles are allowed. Both administrative and multidisciplinary teams have two breaks for meals (15 min and 1 hour) and do not need a substitute to go to the toilet or fill their water bottle whenever they wish. Their work shift varies from 6 to 8.5 hours/day.

### Questionnaire

After approval by the Institutional Review Board, the workers received an invitation by email or by messenger app to participate in an anonymous online questionnaire formulated by the researchers. Participants were provided with written information regarding the study on the first page of the survey, and participation was taken as implied consent. The questionnaire comprised questions regarding demographics and job characteristics, usual daily liquid intake (in cups/day), usual daily urine voiding frequency, and usual urine color according to the urine color chart. Participants also answered if they had (and if they did, with what frequency) any of the following urinary tract symptoms in the last 12 months: burning feeling, frequent or intense urge to urinate, bloody urine, and lower abdomen pain. For analysis, we considered any positive answer as having a urinary tract symptom for comparison with participants with negative responses. The occurrence of reported urinary tract infection (cystitis) episode was evaluated separately. We also asked: At work, can you have something to drink whenever you feel thirsty? At work, do you abstain from having a drink to avoid needing to use the toilet? The five possible answers for both questions were: always, most of the time, sometimes, rarely, and never. We grouped "always" and "most of the time" versus the other three options for analysis.

The participant's perceptions of work facilities regarding the distance to a drinking water source from the workstation, toilet distance, number of toilets available, and toilet hygiene were also investigated. The three possible answers were: it favors hydration, neither favors nor hinders hydration, or hinders hydration. We grouped the first two answers for comparison analysis.

### Statistical analysis

Variables are reported as means and standard deviation or medians and interquartile ranges or as percentages, as appropriate. The t-test was used to compare groups where variables were normally distributed; otherwise, Mann-Whitney test was used. Chi-square or Fisher's exact tests were performed to compare distributions when appropriate. IBM SPSS Statistics for Windows version 21.0 was used to analyze the data. P-values < 0.05 were considered significant.

## Results

Of the 258 invitations sent to the women, we received completed online forms from 179 (69.4%). We excluded forty-six forms due to eligibility criteria. The final sample included 133 female workers (66 nurses and 67 controls). The nurses were younger than the controls and had shorter company time. Among hydration markers, nurses reported a lower total fluid intake. A lower urine void frequency and higher urine color score were reported by nurses but did not differ significantly from controls. Ninety-three percent of controls reported being able to have a drink when thirsty at work always or most of the time compared to less than half of nurses (Table 1).

**Table 1:** Comparisons of the main characteristics and hydration behaviors between nurses and controls (N=133)

	Total (N=133)	Nurses (N=66)	Controls (N=67)	P
Age (years)	36.1± 8.9	34.2 ± 8.8	38.2 ± 8.5	0.005
Company time (years)	5 (3-10)	5 (2-7.5)	6 (3-12)	0.008
Fluid intake (cups/day)	6.6 ± 2.9	6.0 ± 2.6	7.3 ± 3.0	0.01
Urine void frequency (times/day)	5.4 ± 2.1	5.1±2.3	5.7 ± 1.8	0.11
Urine color chart score	3.0 ± 1.2	3.1 ± 1.3	2.9 ± 1.1	0.25
Drinking when thirsty	68%	44%	93%	<0.001
(always/most of the time)				
Abstain liquids to avoid going to the toilet	12%	18%	7%	0.07
(always/most of the time)				

**Figure 1:**
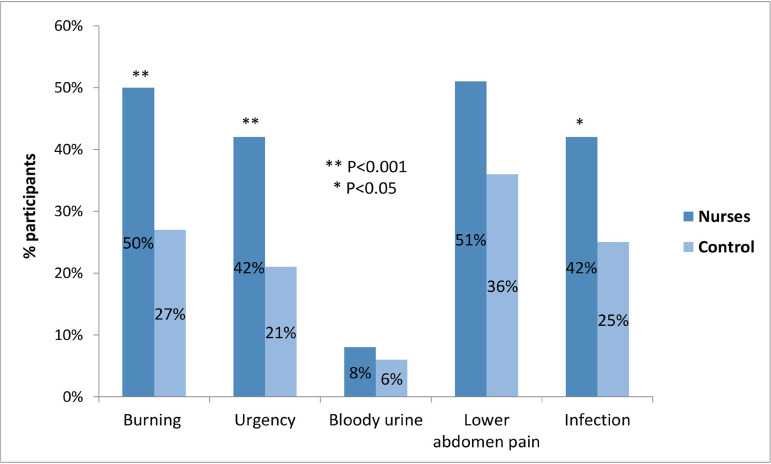
Self-reported prevalence of urinary tract symptoms and infection in the previous 12 months among nurses and controls.

Concerning self-reported urinary symptoms and infection in the previous year, nurses had a higher prevalence of burning feeling, urinary urgency, and infection compared to controls (Figure 1).

Our analysis of the self-reported hydration markers showed that participants that reported at least one symptom (N=70 / 53%) had a lower urine void frequency (5.0±2.3 versus 5.8±1.8 times/day; P=0.04) and higher urine color score (3.3±1.2 versus 2.6±1.1; P=0.002) than participants without symptoms.

**Figure 2:**
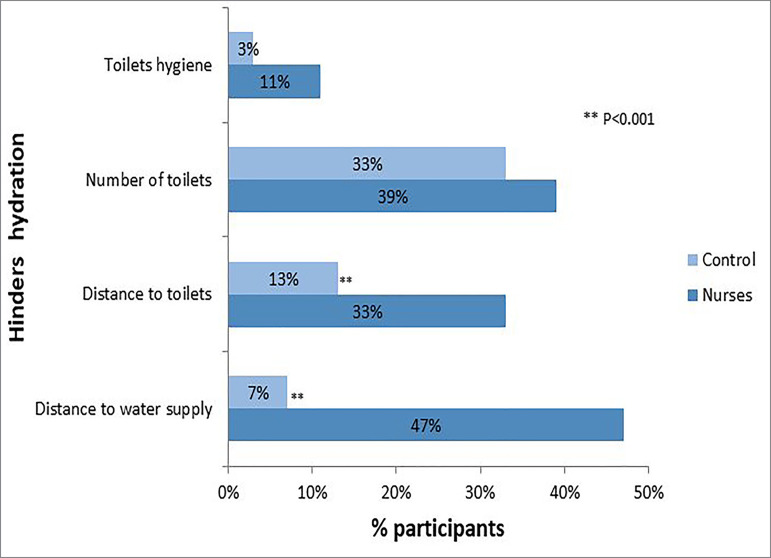
Perceptions of work infrastructure regarding drinking water supply and toilet aequacy in nurses and controls.

Regarding work infrastructure, almost half of nurses (47%) considered the distance to drinking water source a hindrance to hydration compared to only 7% of the control group (P<0.001). The toilet's distance was also considered a barrier by a greater number of nurses (33 versus 13%; P=0.007). Perceptions regarding the number of toilets available and toilet hygiene did not differ between groups (Figure 2).

Analysis of the impact of work environment perceptions of urinary problems showed significant associations only in the nurses group. Participants who considered the drinking water supply a barrier to adequate hydration had a higher prevalence of symptoms (77% versus 54%; P=0.04). A similar result was observed regarding toilet distance. Almost all female nurses (92%) who considered it a barrier had some symptom while only 47% among those who did not have this perception (P<0.001) had symptom. Furthermore, nurses who responded that toilet hygiene hindered hydration had a higher prevalence of infection (86% versus 37%; P=0.03).

## Discussion

The results of this cross-sectional questionnaire survey revealed that dialysis female workers have a lower water intake and a higher prevalence of urinary tract symptoms and infection than other occupations sharing the same environment.

Although water necessity varies according to individual metabolism, climate, and physical activity level, a total intake of 2,700 mL/day from all dietary sources is recommended for adult women^14^. As 20-25% of liquid intake comes from food, a total liquid intake of approximately 2000 mL/day is considered adequate under moderate ambient temperature. Therefore, although the female nurses reported a lower usual liquid intake than controls (~1200 versus ~1450 mL/day), consumption in both groups requires improvement. Other investigations with nurses reported a mean intake of 4 cups in an 8h shift^13^ as well as a total fluid intake below 2000 mL/day in 86% of participants^10^.

Since the participants in the present study share the same infrastructure, the work-related factors possibly linked to the lower liquid intake by nurses besides the lack of water bottles in workstations include the requirement for a substitute when leaving the dialysis rooms, making it challenging to leave the worksite outside of predetermined breaks. Indeed, only 44% of nurses reported being able to drink when thirsty "always" and "most of the time" versus 93% of the control group.

A lower fluid intake leads to more concentrated urine and less frequent urine voiding. Thus, urinary markers are also used to assess hydration status. A 24h-urinary frequency of > 6 voids and urine color score ≤ 3 are associated with better hydration^15^^,^ 16. In our whole population, the mean urinary frequency of 5.4 ± 2.1 and urine color score of 3.0 ± 1.2 reflected the reported low liquid intake. Furthermore, urinary markers (frequency and color) were associated with the symptoms.

Forty-two percent of nurses reported at least one urinary tract infection. Previous investigations of urinary tract infection in nurses used different methods, making it difficult to perform direct comparisons. In a cross-sectional study, Bendtsen found that 16% of nurses had symptoms that could be ascribed to cystitis^11^. Among 636 Chinese nurses that completed a questionnaire, 23% reported a history of urinary tract infection^10^. However, neither study had a control group. Our literature search revealed only one study that also used a control group to compare the occurrence of lower urinary tract symptoms (LUTS) between female nurses and secretaries working in the same hospital; however, that study did not include urinary tract infection. Although there were significant differences in job conditions, the authors reported a similar prevalence of LUTS in both groups^17^.

Although all workers in this study shared the same work infrastructure, their perception regarding the distance to a drinking water supply and bathrooms from their worksite differed significantly. Water supply distance was considered a barrier to proper hydration by 47% of nurses and by only 7% of the controls. We hypothesize that demanding activities combined with not being able to have a water bottle at the worksite was the main reason for this result. Regarding the bathroom distance, 33% of nurses considered it a barrier versus 13% of controls. The inability to leave the dialysis rooms at any time may have influenced this perception. The number of toilets was considered adequate by around two-thirds and hygiene as proper by most participants in both groups.

The perceptions of work infrastructure adequacy were associated with the presence of urinary symptoms and infection only in the nurses group. Those that considered the distance to the water supply or toilet facilities a barrier to adequate hydration showed a higher prevalence of urinary symptoms than nurses with different perceptions (77 versus 54%; P=0.04 and 92 versus 47%; P<0.001, respectively). Furthermore, although only 11% of nurses considered toilet hygiene a hindrance to hydration, 87% of these workers reported one or more urinary tract infection episodes in the preceding year, while the corresponding proportion among nurses who did not perceive toilet hygiene as a hindrance was 37%.

In a Chinese study, toilet hygiene, limited toilet facilities, and inaccessibility were predictors of toilet behaviors in the workplace. The most prevalent unhealthy behavior was delayed voiding^10^.

Pierce et al. (2019)^18^ performed an in-depth qualitative exploration of nurses' and midwives' experiences of urinary symptoms at work through focus group discussions. The participants reported delaying voiding due to a work culture of "patient-first" care at the expense of self-care, relationships in the nursing team, demands of the nursing role, and inadequacy of workplace amenities. The barriers included the distance from the clinical work area, inadequate number of toilets, and reluctance to use amenities with a lack of privacy or hygiene. The storage and voluntary emptying of urine are not just physiological processes but are also influenced by psychological, socio-cultural, and environmental factors. The privacy, safety, cleanliness, and comfort of public toilets are important to many women^19^.

Based on ours and previous findings, strategies to promote better hydration and toileting habits among female workers, especially nurses, are imperative to decrease the risk of urinary problems and their adverse health and work-related consequences. The "patient-first culture" without a proper approach in academic and professional settings have implications of assimilating and acting without critical thinking and analysis of contexts and situations.

We did not find any interventional studies on this topic among nurses; however, positive results were reported among industrial "clean-room" workers. A study in 2002 found that the prevalence of urinary tract infection among these workers was 2.5-fold higher than that among workers in other occupations due to the troublesome process of changing special clothing and cleaning procedures required to leave the worksite to drink water or use toilets^3^. After an intervention that included a health education program with different tools, a significant decrease in the prevalence of urinary tract infection measured by urinalysis (from 9.8 to 1.6%) and significant increases in water intake and urine voiding were observed^20^. A recent randomized controlled trial reinforced the common medical advice of increasing water intake to prevent urinary infection. Authors found a significant reduction in recurrent cystitis in women who had previously consumed low volumes of fluids daily (<1.5 L per day) and increased 24-h urine voiding from 6.0 ± 1.2 at baseline to 8.2 ± 1.2 times after 12 months of intervention^21^.

Our study limitations include using a self-reported questionnaire without clinical confirmation and the lack of control for the frequency of sexual intercourse, an important risk factor for urinary tract infection, especially in women. As with all cross-sectional studies, we were unable to assess causality or temporal relationships. Our main strength was the inclusion of a control group working in the same environment to specifically identify work-related issues in nurses.

In conclusion, dialysis nurses reported a lower fluid intake and higher prevalence of urinary tract symptoms and urinary tract infection than administrative and multidisciplinary staff. Also, nurses who perceived environmental barriers to adequate hydration had a higher prevalence of urinary problems. Our results corroborate previous findings and highlight the importance of investigations to identify barriers that discourage adequate hydration and its adverse consequences. Interventions involving policymakers, healthcare organizations, and health education focusing on the importance of self-care can potentially decrease urinary problems in these populations.
